# *ROGDI*-Related Disorder Resulting from Disruption of Complex Interactive Neuro-Dental Developmental Networks: A Review and Description of the First Missense Variant

**DOI:** 10.3390/genes16101207

**Published:** 2025-10-14

**Authors:** Sopio Gverdtsiteli, Trine Bjørg Hammer, Xenia Hermann, Noemi Becser Andersen, David Ros-Pardo, Iñigo Marcos-Alcalde, Paulino Gómez-Puertas, Alan Henry Brook, Asli Silahtaroglu, Zeynep Tümer

**Affiliations:** 1 Department of Clinical Genetics, Copenhagen University Hospital, Rigshospitalet, DK-2100 Copenhagen, Denmark; sgve@filadelfia.dk (S.G.); trine.bjoerg.hammer@regionh.dk (T.B.H.); zeynep.tumer@regionh.dk (Z.T.); 2 Resource Center for Rare Oral Diseases, Copenhagen University Hospital, Rigshospitalet, DK-2100 Copenhagen, Denmark; xenia.hermann@regionh.dk; 3 The Epilepsy Clinic, Department of Neurology, Copenhagen University Hospital, Rigshospitalet, DK-2100 Copenhagen, Denmark; noemi.becser.andersen.01@regionh.dk; 4 Molecular Modeling Group, Centro de Biologia Molecular Severo Ochoa (CBM, CSIC-UAM), E-28049 Madrid, Spain; dros@posta.unizar.es (D.R.-P.); imarcos@cbm.csic.es (I.M.-A.); pagomez@cbm.csic.es (P.G.-P.); 5 Adelaide Dental School, The University of Adelaide, Adelaide, SA 5000, Australia; alan.brook@adelaide.edu.au; 6 Department of Cellular and Molecular Medicine, Faculty of Health and Medical Sciences, University of Copenhagen, Blegdamsvej 3, DK-2200 Copenhagen, Denmark; 7 Department of Clinical Medicine, Faculty of Health and Medical Sciences, University of Copenhagen, Blegdamsvej 3, DK-2200 Copenhagen, Denmark

**Keywords:** *ROGDI*-related neurodevelopmental and dental disorder, Kohlschütter–Tönz syndrome, amelocerebrohypohidrotic syndrome, amelogenesis imperfecta, ROGDI, complex interactive developmental networks

## Abstract

*ROGDI*-related neurodevelopmental and dental disorder (*ROGDI*-RD), also known as Kohlschütter–Tönz syndrome (KTZS, MIM #226750), is a rare condition characterized by developmental abnormalities affecting both the central nervous system (CNS) and the dentition. These phenotypes highlight the role of complex gene–environment interactions and developmental networks shared by the nervous and stomatognathic systems, both of which originate mostly from neural crest-derived cells. In this review, we analyze clinical and genetic data from 54 previously reported *ROGDI*-RD patients to better define the phenotypic spectrum of the disorder. Most of the reported cases harbor protein-truncating variants. Here, we also present the first description of a patient carrying a missense variant in *ROGDI* atypical leucine zipper gene, *ROGDI* in trans to a frameshift variant. This individual presented with tooth agenesis—a dental anomaly not previously associated with the syndrome—alongside classic neurological and dental enamel features, suggesting that the phenotypic spectrum of *ROGDI*-RD may be broader than currently recognized. Using a complexity and network science framework, we discuss how dysregulation in multilevel, interacting developmental systems may explain the pleiotropic features of *ROGDI*-RD. Our findings underscore the importance of early, interdisciplinary clinical evaluation in patients with neurodevelopmental symptoms and enamel defects. As enamel phenotypes such as amelogenesis imperfecta are heterogeneous, comprehensive genomic analyses and collaborative clinical approaches are essential for accurate diagnosis and improved care.

## 1. Introduction

Syndromes with pleiotropic effects enhance our understanding of the underlying factors and their interactions. *ROGDI*-related disorder (*ROGDI*-RD), also known as Kohlschütter–Tönz syndrome (KTZS, MIM **#** 226750), is one such syndrome affecting both the dentition and the central nervous system. *ROGDI*-RD is caused by homozygous or compound heterozygous variants in *ROGDI*, which encodes a leucine-zipper protein (ROGDI atypical leucine zipper).

Development of the dentition and the orofacial complex is under the influence of complex adaptive systems involving genetic, epigenetic, and environmental factors [1]. Similar interactions are also implicated in the development of the central nervous system (CNS). In both tissues, these processes occur within a multilevel, complex interacting network [2]. Both the dentition and the CNS originate embryologically from neural crest-derived cells.

In this review, we examine 54 published *ROGDI*-RD patients to clarify the clinical spectrum of the syndrome. We also analyze previously reported genetic variants—mainly protein-truncating variants—and present, to our knowledge, the first missense variant in *ROGDI* identified in a patient with tooth agenesis, a developmental dental anomaly not previously associated with the syndrome. To deepen our understanding of its etiology and phenotype, we explore these aspects using the framework of complexity and network sciences, seeking to uncover underlying mechanisms and broader clinical insights.

## 2. The Disease and the Protein

### 2.1. ROGDI-Related Neurodevelopmental and Dental Disorder (ROGDI-RD)

*ROGDI*-RD is an autosomal recessive disease characterized by global developmental delay with regression, epilepsy, and amelogenesis imperfecta causing yellow or brown discoloration of the teeth.

*ROGDI*-RD is one of the rare ectodermal dysplasia syndromes, and it is also considered an epileptic encephalopathy as the intellectual impairment is likely to be linked to the severity of the epilepsy. There is variable expressivity in the spectrum and the severity of symptoms, even within the same family, regarding the facial features, age at onset of seizures, developmental status prior to seizures, seizure type, response to treatment, discoloration of the teeth, and neurological and cognitive symptoms [3].

Recent studies have identified pathogenic variants in *SCL13A5* and *SATB1* as causes of neurodevelopmental disorders accompanied by dental anomalies. Although these genes have been grouped under the broader category of conditions referred to as Kohlschütter–Tönz syndrome, their inheritance patterns and particularly the dental phenotypes differ from those observed in the *ROGDI*-related form of the disease [4,5,6]. While this may reflect genetic heterogeneity, it is also plausible that these genes are related to distinct conditions affecting the same organ systems along the oral–brain axis but with overlapping yet divergent clinical features, especially given that *SCL13A5* and *SATB1* do not have roles in V-ATPase function. For this reason, we propose using the term *ROGDI*-related disorder rather than the umbrella term Kohlschütter–Tönz syndrome. Gene-based nomenclature facilitates more accurate phenotypic comparisons and may also support future reclassification efforts, as additional syndromes involving both dental and CNS symptoms are identified.

### 2.2. ROGDI Expression and Function

*ROGDI* is highly expressed in the adult human brain and the spinal cord, while expression is much lower in the fetal brain [7,8]. It has been shown that ROGDI has a pre-synaptic localization in neurons and is proposed to be involved in the regulation of exocytosis in developing nerve endings and ameloblasts [9].

The main function of ROGDI is still elusive, but it is suggested to have a role in keeping the pH acidic within the organelles and degradation of proteins [10]. ROGDI is the human homolog of Rav2 of the yeast RAVE complex. It is essential for efficient reassembly of the V-ATPase, which is crucial for vacuolar function and cellular pH homeostasis. Human ROGDI can partially rescue the growth defect of yeast rav2Δ mutants, supporting a conservation of function [10,11].

The *ROGDI*^−/−^ knockout mouse model develops *ROGDI*-RD-like symptoms such as epilepsy, memory impairment, and amelogenesis imperfecta with severe enamel hypomineralization, also suggesting a role for ROGDI in the regulation of lysosomal acidification due to decreased V-ATPase activity caused by impaired Rabconnectin-3 complex [10,11]. ROGDI is a prominent member of the mammalian Rabconnectin-3 complex together with DMXL1, DMXL2, and WDR7. In this complex, ROGDI is the central linker bridging the proteins Rabconnectin-3a and Rabconnectin-3b encoded by DMLX2 and WDR7, respectively. The combination of neurological and tooth phenotypes in *ROGDI*-RD patients is consistent with ROGDI bridging Rabconnectin-3a and Rabconnectin-3b subunits which show tissue-specific enrichment relevant to these affected organs [10,11].

Variants of the genes encoding the members of the Rabconnectin-3 complex, i.e., V-ATPases, DMXL2, and WDR7, result in epilepsy, intellectual disability, autism spectrum disorder, or enamel defects [10,12]. Furthermore, both Rogdi and Dmxl2 are downregulated in the trisomic synaptic fractions of the Ts65Dn mouse model for Down syndrome [13].

## 3. Review of the Patients with *ROGDI*-RD Including a New Case

Since the initial description of *ROGDI*-RD in 1974, 54 individuals with homozygous or compound heterozygous pathogenic *ROGDI* variants have been identified [3,14]. To date, all the sequence variants reported in *ROGDI*-RD patients are predicted to be null variants, suggesting loss of function as the disease mechanism [7,15]. We recently identified compound heterozygous *ROGDI* variants in a patient, one of which is a missense variant. To the best of our knowledge, this is the first report of a disease-causing missense variant in *ROGDI.*

### 3.1. Clinical Features of the New Case

A 28-year-old man with intellectual disability, autism, attention-deficit hyperactivity disorder (ADHD), and epilepsy was referred to the Department of Clinical Genetics, Rigshospitalet for genetic diagnosis. He was born to non-consanguineous parents after an unremarkable pregnancy. The birth weight was 3540 g and birth length 54 cm. During infancy, he exhibited persistent irritation and poor eye contact. Motor milestones were slightly delayed: he achieved head control at 3.5 months, sat without support and began crawling at 9 months, and walked by 2 years of age.

He experienced his first generalized tonic–clonic seizure (GTCS) at 11 months of age. Subsequently, he had both febrile and afebrile seizures. From the age of 6 years, he began experiencing focal impaired awareness seizures. Seizures persisted despite the trial of conventional antiseizure medications (ASMs). As the patient had multifocal epilepsy, surgical treatment could not be offered. At the age of 16, a vagus nerve stimulator (VNS) was implanted, resulting in a reduction in GTCS frequency, but it did not have a clear effect on focal seizures. The patient is treated with ASMs, eslicarbazepine, phenobarbital, and rufinamide, alongside the VNS. There is no specific treatment available for this syndrome, and the patient’s epilepsy is treated with the principles of treating refractory epilepsy in general. The patient was treated with methylphenidate for ADHD in his childhood. Currently, risperidone is used for behavioral disorders, with good response.

Physical examination at the age of 23 revealed retrognathia, downward-pointing columella, low-set ears, and scoliosis. He spoke in simple two–three-word sentences and used picture-based communication tools. ADHD and behavioral disorder in the form of verbal and physical aggression, mild self-harm, and restlessness were also noted. Brain magnetic resonance imaging did not show any pathological changes. Audiometric, ophthalmologic, and abdominal ultrasound examinations were all within normal limits.

**Figure 1:**
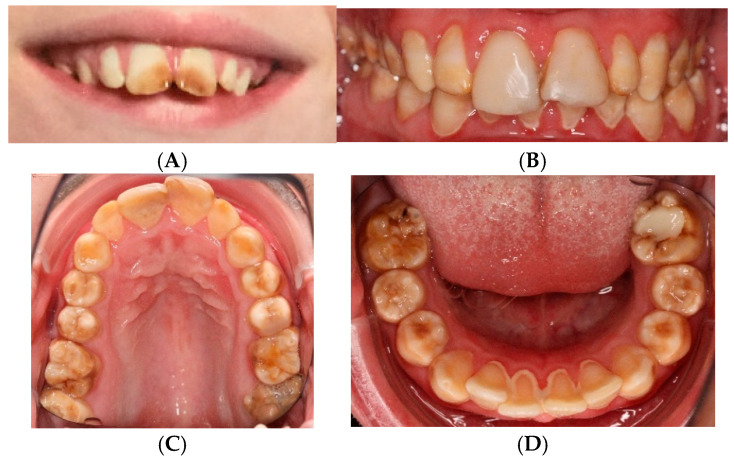
(**A**) Intraoral photo at the age of 10 years (before composites were made for 11 and 21) shows brown color and distinct opacity due to hypomature amelogenesis imperfecta. (**B**–**D**) Intraoral photo at the age of 28 shows composites of 11 and 21, brown and yellow opacity of all teeth, and calculus in the lower front.

**Figure 2:**
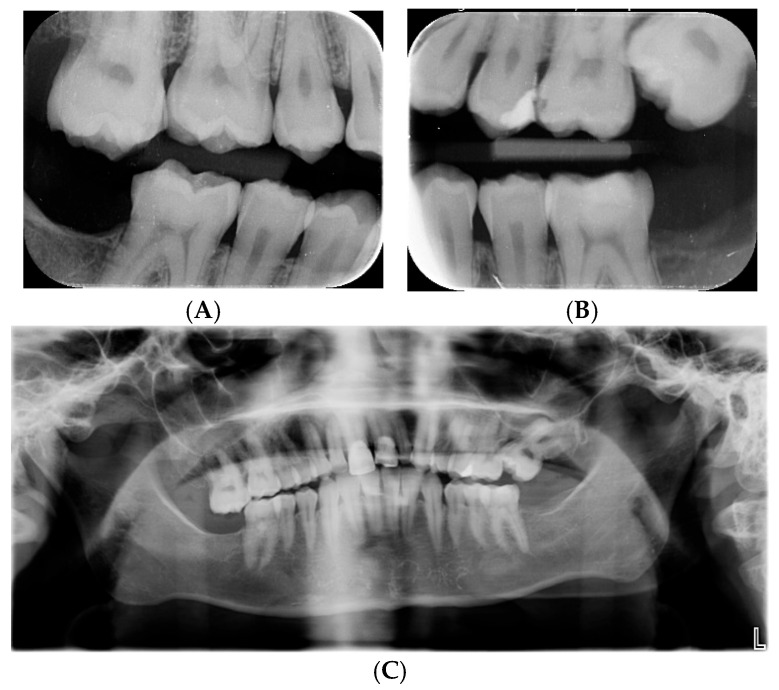
(**A**,**B**) Bitewings radiograph at the age of 18 years shows caries 26 and ectopic 27, yet no resorption of 26. Low contrast between enamel and dentin is observed. (**C**) Panoramic radiograph at the age of 28 years shows movement artifact as well as an ectopic 27 causing resorption distally on 26. Teeth 18 and 28 are congenitally absent, but 37, 38, 47, and 48 were removed at the age of 20 due to ectopic positioning and resorption.

As the teeth phenotype was not among the referral symptoms and one of the variants was a missense variant, the referring clinician was contacted to confirm the dental findings. Clinically, the permanent teeth showed lusterless enamel with white, yellow, and brown opacities. The teeth were crowded, with an overjet of 12 mm, bilateral distal occlusion, and a deep incisor overbite measuring 5 mm. Teeth 11 and 21 had composite restorations (Figure 1). Tooth 27 was ectopically positioned and caused resorption of tooth 26, as seen on radiographs for all teeth. Teeth 18 and 28 were congenitally absent. Teeth 38, 37, 47, and 48 were removed under general anesthesia at the age of 20 due to ectopic positioning of 38 and 48 leading to pathological resorption of the adjacent teeth 37 and 47 (Figure 2). The contrast between enamel and dentin on the radiographs was markedly reduced, indicating poor enamel mineralization. The combined dental, clinical, and radiographic findings are compatible with hypomature amelogenesis imperfecta together with tooth agenesis and eruption defects. See Box 1 for explanation of different types of mineralization defects.

### 3.2. Description of the Genetic Finding

Trio exome sequencing revealed two compound heterozygous variants in *ROGDI* (NM_024589.3): c.218del, p.(Gly73Valfs*3) and c.260T>C, p.(Val87Ala).

The pathogenicity of the two *ROGDI* variants was assessed according to ACMG/AMP (American College of Medical Genetics and Genomics/Association for Molecular Pathology) criteria (https://www.clinicalgenome.org/working-groups/sequence-variant-interpretation/ (accessed on 1 August 2025).

The c.218del, p.(Gly73Valfs*3) variant, inherited from the father, is predicted to lead to frameshift and premature stop of translation by altering the reading frame. It is not reported in the Genome Aggregation Database (gnomAD v.4.1.0; https://gnomad.broadinstitute.org/ (accessed on 1 August 2025)) or the ClinVar database (https://www.ncbi.nlm.nih.gov/clinvar/ (accessed on 1 August 2025)). The variant is classified as pathogenic (PVS1_VSt; PM2_Su; PP4_Mo). The maternally inherited missense variant c.260T>C, p.(Val87Ala) is reported twice (allele frequency of 0.000001733) in heterozygous form in gnomAD and is not present in ClinVar. However, in ClinVar a different nucleotide change affecting the same amino acid, p.(Val87Gly), is reported in a patient with amelocerebrohypohidrotic syndrome, which was a term used to define the same syndrome in the early days. The REVEL score of the variant described in this paper is 0.344, which does not suggest pathogenicity, but the AlphaMissense prediction, which also uses structural representations from AlphaFold2, gives a score of 0.681, which suggests the variant to be likely pathogenic. The variant is classified as a variant of unknown significance using REVEL (PM2_Su; PM3_Mo; PP4_Mo) but as likely pathogenic using AlphaMissense scoring (PM2_Su; PM3_Mo; PP3_Su; PP4_Mo). Uncertainty in pathogenicity prediction remains an inherent challenge in clinical diagnosis, which will likely diminish as more cases are reported, and predictive AI tools continue to improve.

### 3.3. Dynamic Computational Modeling of the Val87Ala Variant

Structural modeling of the wild-type (WT) ROGDI and the Val87Ala variant (UniprotKB id: ID: Q9GZN7) were performed in both monomeric and dodecameric forms using the crystal structure of human ROGDI (PDB: 5XQL; [16]) as a template. Once modeled, the structures were simulated over a period of 300 ns of unrestrained molecular dynamics (MD) simulation using the Amber18 package (https://ambermd.org (accessed on 1 August 2025); University of California-San Francisco, CA, USA), essentially as previously described [17]. Trajectories were analyzed using cpptraj [18] and VMD [19].

**Figure 3:**
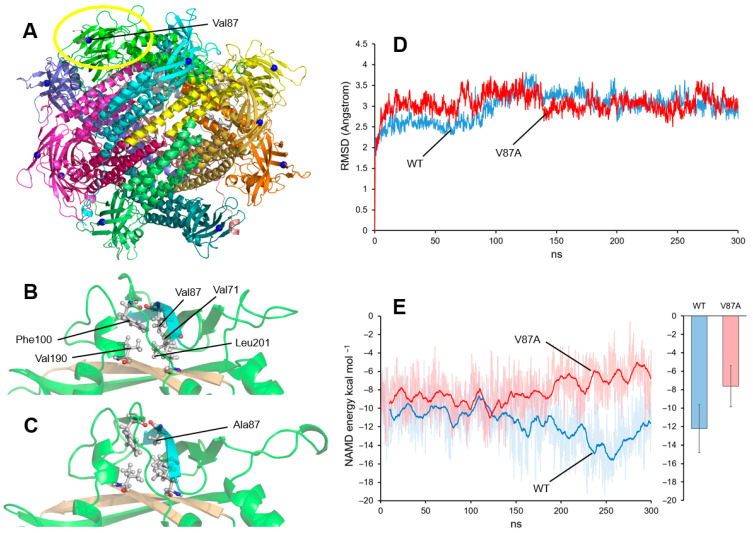
Molecular dynamics analysis of ROGDI Val87Ala variant. (**A**) Structure of the human ROGDI dodecamer. The position of the alpha-carbon of the Val87 is shown (blue spheres). The yellow circle indicates the position of the structures shown in (**B**) and (**C**). (**B**) Structure of the domain around Val87 after 300 ns of unrestrained simulation. The positions of Val71, Phe100, Val190, and Leu201 are indicated in the same hydrophobic cluster as Val87. The beta sheet where Val87 is located is in blue and the opposite sheets with Val190 and Leu201 are in ocher. (**C**) Structure of the domain around the Ala87 after 300 ns of unrestrained simulation. (**D**) Root-mean-square deviation (RMSD) values were measured along molecular dynamics trajectories (300 ns) of the WT (blue) and Val87Ala (red) protein monomer. (**E**) NAMD interaction energy between the opposing beta sheets (blue and ochre in (**B**) and (**C**)). Left: continuous energy values corresponding to WT (blue) and Val87Ala (red). The moving average (calculated every 0.5 ns) of the energy values is highlighted to facilitate visualization. Right: mean +/− STD of the energy values shown in the left plot between nanoseconds 100 and 300.

In the simulation performed with the dodecamer (shown in Figure 3A), no significant changes were observed in the protein structure of Val87Ala compared to the WT). To better detect potential differences in an environment less constrained by the whole polymer structure, a simulation was subsequently performed using only the protein monomer.

Figure 3B shows the result of a simulation of the WT monomer after 300 ns of unrestrained molecular dynamics. Val87 is part of a cluster of hydrophobic amino acids that maintains the local structure of a domain formed by several beta sheets. Val87 interacts with Val71 and Phe100, which are located in beta strands parallel to the one containing Val87, and with Val190 and Leu201, which are located in the opposite beta sheet. In the mutant protein (Figure 3C), the Ala87 amino acid also interacts with the same residues, although the interaction appears to be slightly less compact due to the smaller size of the alanine compared to valine. This difference does not translate into a change in the monomer’s overall structure, as indicated by the nearly equivalent RMSD (root-mean-square deviation) values of the WT and Val87Ala throughout the entire molecular dynamics trajectory (Figure 3D).

The nonbonding energy contributions of the interaction between the beta sheet containing the variant residue (blue in Figure 3B,C) and the beta sheets containing Val190 and Leu201 on the opposite sheet (ochre in Figure 3B,C) were evaluated using the NAMD Energy Plugin of VMD (NAMD v.2.14; Philips et al., 2005) [20]. Figure 3E shows the NAMD interaction energy values between these structures throughout the entire trajectory, as well as a calculation of the average values starting from nanosecond 100 of the simulation (to allow for a prior equilibration time). With this simulation, a slight difference between the energy values of WT and Val87Ala was detected.

Although these are not very significant differences, higher (i.e., less favorable) energy values can be observed in the case of the Val87Ala variant. This difference does not seem to be enough to cause structural changes in the mature protein, but an effect during protein folding cannot be ruled out. This could perhaps result in a misfolded mature protein or a smaller amount of correct protein in the cell. One of these molecular-level effects could be responsible for the phenotypic effect observed in the patient.

## 4. Review of the Phenotype and Genotype of *ROGDI*-RD Patients

### 4.1. The Clinical Features

To date, 54 individuals with *ROGDI* variants have been reported [7,8,15,20,21,22,23,24,25,26,27,28,29,30,31,32]. The clinical features of the published cases and the present case are listed in Table 1. The review of the clinical characteristics revealed that all the patients had developmental pathologies both in the brain and teeth. Developmental delay was present before the onset of epilepsy in 42% of the patients, while in 58% of the individuals the developmental delay was observed following the start of the epileptic seizures. Speech delay was a prominent feature, with 67% being non-verbal, and most patients (76%) had profound/severe intellectual disability. Epilepsy was reported in all the individuals, and median age at seizure onset was 9 months (range: 1–48 months). Seizures were sensitive to fever in 20% of the individuals either at onset or at follow-up. Epilepsy proved to be drug-resistant in 25% of cases. Dental abnormalities were also a consistent feature in 100% of the individuals, presenting with enamel defects. The appearance of these defects was often reported as consistent with hypomature amelogenesis imperfecta. See Box 1 for explanation of pathology in amelogenesis.

### 4.2. ROGDI Variants and the Phenotype

All the *ROGDI* variants identified to date are predicted to be protein-truncating variants, possibly resulting in loss of protein function. In this study, we expand the molecular spectrum by presenting a missense variant in trans with a frameshift variant. The phenotype of the present case is fully compatible with *ROGDI*-RD, suggesting that the missense variant is likely to have a loss-of-function effect.

This case exemplifies the importance of having both the detailed phenotype and the genotype for the definitive diagnosis. Initially, only seizures and developmental delay were reported as the symptoms of the patient. However, upon re-examining the patient’s medical history after identification of the *ROGDI* variants, one of which was a VUS, it was found that during childhood he had tooth discoloration and enamel defects, which were treated. This study underlines the importance of detailed clinical information for the evaluation of the sequence data. Given the limited number of reported patients and the genetic homogeneity among them, meaningful statistical meta-analyses or genotype–phenotype correlations are not yet feasible; however, future studies including more genetically diverse cases may help to address this limitation.

### 4.3. Complex Interactive Neuro-Dental Developmental Networks and ROGDI-RD

While dentin and dental pulp originate from the neural crest, enamel is derived from neuroectoderm-like structures in the brain [33,34]. Tooth development, which starts with the formation of the dental lamina at 6 weeks in utero, is a continuous process, with critical time windows. Mineralization of primary incisors commences at around 4 months in utero, and the full primary dentition erupts by 3 years of age. Permanent second molars develop from 3 years to 14–16 years, while the development of permanent third molars is completed by the age of 18–25 years [33,35].

The human brain’s development is also profound from the second trimester of the fetal period starting with the growth of initial axons, until young adulthood. Developmental changes in neuronal connectivity reflect the social, cognitive, and motor skills of the individual during this period. Interruption of development increases the risk for neurodevelopmental disorders such as autism spectrum disorder, attention-deficit hyperactivity disorder, and schizophrenia. Sensitive periods for brain and tooth development are suggested to coincide [33,36].

While the question is still open about the extent of interplay between these two dynamic developmental systems of dentition and brain, the observation that enamel defects are quite common among patients with congenital CNS disorders supports the interaction between these two networks.

Many key neuropeptides involved in brain development and function, along with their receptors—including serotonin, melatonin, and circadian rhythm genes—are expressed by ameloblasts. Additionally, some markers specific to glial cells are expressed in the dental pulp, confirming the connection. All these factors are shown to have the ability to modulate enamel formation, and knockout animal models have exhibited teeth malformations [33,34,37,38].

In a review of enamel-related phenotypes, Wright [6] listed 18 syndromes—including *ROGDI*-RD and other forms of Kohlschütter–Tönz syndrome—that are characterized by enamel and CNS abnormalities. Among these, two syndromes—intellectual developmental disorder with speech delay, dysmorphic facies, and T-cell abnormalities (associated with *BCL11B* variants) and oculoskeletodental syndrome (due to *PIK3C2A* variants)—also present with tooth agenesis for the patient presented in this study.

Given that different mechanisms regulating tooth number and enamel formation function at different stages of odontogenesis, it remains an open question whether this is a pleitropic effect of a single gene variant or whether the tooth agenesis is an independent event since it has a high prevalence and multifactorial etiology in the general population [39,40]. Nevertheless, we propose that tooth agenesis should be systematically evaluated in future patients, as this may help determine whether it could serve as a supportive diagnostic criterion for *ROGDI*-RD.

The *ROGDI*-RD case illustrated in Wright [6] exhibited hypomineralized enamel defects affecting the primary dentition. Since primary incisors typically erupt between 6 and 15 months of age, the late onset of epilepsy and regressive CNS symptoms during this period may reflect a critical window of interaction between the complex neuronal and dental developmental pathways. Therefore, simultaneous early screening for dental and neurological symptoms could facilitate earlier diagnosis of *ROGDI*-RD, enabling timely intervention and potentially mitigating disease progression. A full preventive dental care program should be established for the patients where mineralization of the hypomineralized enamel could be undertaken with fluoride and/or casein phosphopeptide amorphous calcium phosphate agents from a very young age and a restorative treatment can be provided, when necessary.

Box 1 **Tooth development and terms used for enamel pathology**

Tooth development follows the sequence of initiation, morphogenesis, differentiation, and mineralization [35,40]. The formation of enamel, amelogenesis, is controlled by differentiated and highly specialized ameloblasts which deposit a matrix that becomes progressively mineralized [6]. Disruption of matrix deposition by genetic or environmental factors leads to reduced enamel thickness termed enamel hypoplasia. When mineralization is affected, the enamel is formed in full thickness but is hypomineralized in severe cases while in milder cases characterized as hypomature [6].

## 5. Conclusions

The present study is the first report of a novel missense variant in trans to a frameshift variant in *ROGDI* and underlines the importance of a thorough clinical investigation in early diagnosis. Since amelogenesis imperfecta is a very broad enamel phenotype [22], extensive sequencing approaches and multidisciplinary efforts could enable a thorough stratification of the patient groups and ensure that the oral symptoms of patients with brain-related conditions, such as epilepsy or cognitive delay, are appropriately recognized and addressed. The interaction between the nervous and stomatognathic systems is a complex, bidirectional process mediated by different signaling molecules and cellular interactions. Although the precise function of ROGDI remains incompletely understood, its role in V-ATPase assembly and lysosomal acidification suggests that modulating organelle pH or enhancing V-ATPase activity could be explored as potential therapeutic strategies. Preclinical models, such as the *Rogdi*^−/−^ mouse, may provide platforms to test such interventions. Further research and a multidisciplinary approach are needed to fully elucidate the underlying mechanisms, particularly at the molecular level, to develop effective prevention and treatment strategies.

## Figures and Tables

**Table 1:** Frequencies of the clinical features in previously reported *ROGDI*-RD patients and comparison with the present case. Denominators specify the number of individuals for whom that specific information is available.

Category	Published Cases (% of the Symptom)	Clinical Features of the Present Case
General information		
Male	30/54 (56%)	
Female	24/54 (44%)	+
Age at examination	1.5–18 years	28 years
Pregnancy and Delivery		
Normal	23/33 (70%)	+
Abnormal	10/33 (30%)	
Development		
Developmental delay	54/54 (100%)	+
Delay from birth	14/33 (42%)	+
Delay after seizure onset	19/33 (58%)	
Mild/moderate intellectual disability	5/21 (24%)	+
Profound/severe intellectual disability	16/21 (76%)	
Speech delay	11/33 (33%)	
Absent speech	22/33 (67%)	+
Ability to walk unaided	22/29 (76%)	
Epilepsy	51/51 (100%)	
Median age of onset (range)	9 months (1–48 months)	11 months
Febrile seizures	10/51 (20%)	+
Refractory to treatment	13/51 (25%)	+
Dental features		
Amelogenesis imperfecta/teeth discoloration	54/54 (100%)	+
Additional features		
Normal head circumference	11/18 (61%)	
Microcephaly	7/18 (38%)	+
Dysmorphic features	11/54 (20%)	+
Neuroimaging		
Cerebral atrophy	8/21 (38%)	
Enlarged ventricles	7/21 (33%)	
Cerebellar hypoplasia	3/21 (14%)	

## Data Availability

All the data obtained in this study except for full WGS result have been included in the paper. We do not have consent to share the genomic information.

## Abbreviations

ROGDI-RD: *ROGDI*-related neurodevelopmental and dental disorder; Kohlschütter–Tönz syndrome.
